# Serotonin syndrome following intentional sertraline overdose with recent duloxetine exposure: Diagnostic considerations

**DOI:** 10.1192/j.eurpsy.2026.11331

**Published:** 2026-07-31

**Authors:** N. A. Linero Ríos, M. E. Arévalo Gil

**Affiliations:** 1 Department of Psychiatry, Hospital Central de la Defensa “Gómez Ulla”; 2 Department of Psychiatry, Hospital Universitario “Infanta Leonor”, Madrid, Spain

## Abstract

**Introduction:**

Serotonin syndrome (SS) is a potentially life-threatening condition caused by excessive serotonergic activity. It typically results from overdose or drug interactions involving serotonergic antidepressants. SS is characterized by a triad of cognitive/behavioral (agitation, confusion), autonomic (hyperthermia, diaphoresis, hypertension, tachycardia), and neuromuscular symptoms (tremor, hyperreflexia, clonus, dystonia). Complications can include rhabdomyolysis, seizures, multi-organ dysfunction, and death. Sequential or combined exposure to SSRIs and SNRIs may increase risk, even after short intervals between treatments.

**Objectives:**

To describe the clinical presentation and management of SS following sertraline overdose in a young adult recently treated with duloxetine, and to discuss the possible pharmacodynamic contribution of recent SNRI exposure.

**Methods:**

A detailed case analysis and literature review were conducted. A 20-year-old male with a history of emotional instability and multiple self-harm attempts intentionally ingested approximately 2 g of sertraline, 10 mg of clorazepate, and 60 mg of duloxetine ten days after hospital discharge, during which duloxetine had been prescribed at 60 mg/day following discontinuation of sertraline.

**Results:**

At presentation, the patient exhibited hypertension, tachycardia, diaphoresis, and mild neuromuscular signs including jaw dystonia and hyperreflexia. Within 24 hours, symptoms progressed to ocular and patellar clonus, mydriasis, hyperthermia, and laboratory abnormalities including elevated creatine kinase, hepatic enzyme alterations, and indirect hyperbilirubinemia. Alarms for possible systemic complications prompted transfer to the intensive care unit (ICU) for close monitoring. Supportive care and benzodiazepines were administered. During ICU stay, neurological signs stabilized, vital signs normalized, and no additional organ dysfunction occurred. Symptoms resolved completely within 48 hours. The case illustrates that recent duloxetine exposure may have contributed to the severity of SS, highlighting the potential cumulative serotonergic effect even after short washout periods.

**Conclusions:**

This case emphasizes the importance of high clinical suspicion for SS in patients presenting with overdose, particularly when recent serotonergic therapy has been administered. Early recognition of neuromuscular and autonomic signs is critical to prevent severe complications. Clinicians should carefully consider recent pharmacological history during transitions between antidepressants. Timely supportive management, monitoring in intensive care when indicated, and cautious benzodiazepine use are associated with favorable outcomes. Awareness of such interactions can improve safety in psychiatric emergency settings and guide preventive strategies (Boyer et al. N Engl J Med. 2005; 352:1112–1120).

**Disclosure of Interest:**

None Declared

